# A sunflower seed foreign body aspiration misdiagnosed as pneumonia in a child after tetralogy of Fallot operation: A case report

**DOI:** 10.1002/ccr3.6914

**Published:** 2023-02-05

**Authors:** Sang Ngoc Nguyen, Tai Trong Vu, Bach Hoang, Ha Thai Nguyen, Chuc Van Dang

**Affiliations:** ^1^ Haiphong University of Medicine and Pharmacy Haiphong Vietnam; ^2^ Haiphong Children's Hospital Haiphong Vietnam; ^3^ Hanoi Medical University Hanoi Vietnam

**Keywords:** children, foreign body aspiration, sunflower seed

## Abstract

Approaching a child with dyspnea, coughing, and wheezing, doctors must consider a foreign body in the respiratory tract, and bronchoscopy should be performed. This report described a child having sunflower seed aspiration late‐ and misdiagnosed as pneumonia. He failed with antibiotics therapy. The patient underwent a bronchoscopy, and then the seed was found and entirely removed.

## Graphical abstract

If a child has dyspnea, coughing, and wheezing, it is important to evaluate the possibility of foreign body aspiration, such as sunflower seed. If a foreign agent is present, bronchoscopy is an effective method for removing foreign objects.

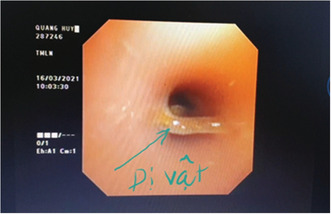

## 1 INTRODUCTION

Foreign body aspiration is a public health issue in many nations, as evidenced by a number of recent publications.^1^, ^2^, ^3^ Detecting foreign body aspiration remains challenging for physicians since it can present symptoms similar to many more prevalent diseases in some cases, prompting clinicians to make an inaccurate diagnosis. Children with foreign body aspirations can recover successfully after discovering and removing foreign objects, thanks to advances in diagnostic imaging. Interventional bronchoscopy, typically performed by surgeons or ENTs, is a standard approach for removing foreign items in many nations.^4^, ^5^

Foreign body aspiration is an emergency, as described in numerous studies,^1^, ^6^ and if we do not act appropriately, the patient may have acute respiratory failure and die. The aspiration of a sunflower seed following a tetralogy of Fallot procedure in a child misdiagnosed with severe pneumonia has also not been documented. In this report, we describe a 9‐month‐old child hospitalized due to the aspiration of sunflower seeds following a tetralogy of Fallot procedure at Haiphong Children's Hospital in Vietnam.

## 2 CASE PRESENTATION

**FIGURE 1:**
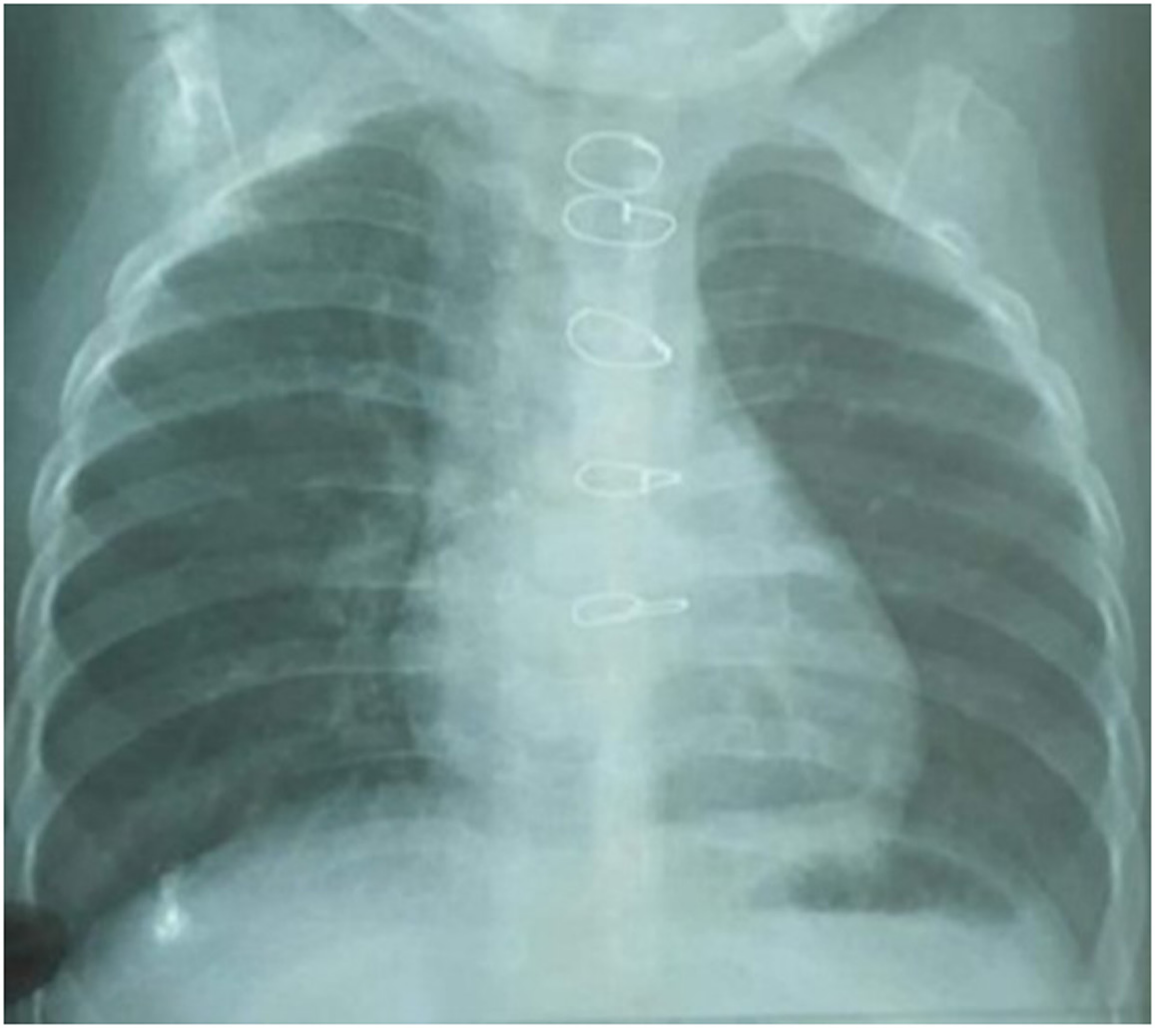
The patient's chest X‐ray.

A 9‐month‐old male infant was admitted to the hospital with cough, dyspnea, and wheezing. His medical history showed he had surgery to treat the tetralogy of Fallot. Upon admission to the hospital, the patient was conscious but had chest contractions and cyanosis. His family did not notice any special events that had happened to him recently. The child weight 8.5 kg, his respiratory rate was 50 breaths/min, his pulse rate was 110 beats/min, his SpO_2_ was 94%, and his temperature was 37.0°C. Chest examination shows a sternal scar, a steady heart rate of 115 bpm, no abnormal heart sounds, and the right lung has wheezing and crackles sounds compared to the normal left lung. His complete blood count showed that the number of white blood cells was 13.4 × 10^9^/L, neutrophils accounted for 15.4%, and serum C‐reactive protein (CRP) was 6 mg/L. Chest X‐ray showed no abnormal images in the bronchi (Figure 1). An echocardiogram found mild pulmonary valve stenosis, and the interventricular septum was closed.

The patient was diagnosed with pneumonia, administered Augmentin for 2 days, and then switched to intravenous ceftazidime for the following 2 days, but the symptom continued to persist. He was eventually given intravenous meropenem and amikacin for the following 7 days, but he failed to recover. The patient continued to cough and wheeze; his respiratory rate was 55 breaths/min, and his SpO_2_ indication was 93%. His nasopharyngeal culture resulted in a negative response.

**FIGURE 2:**
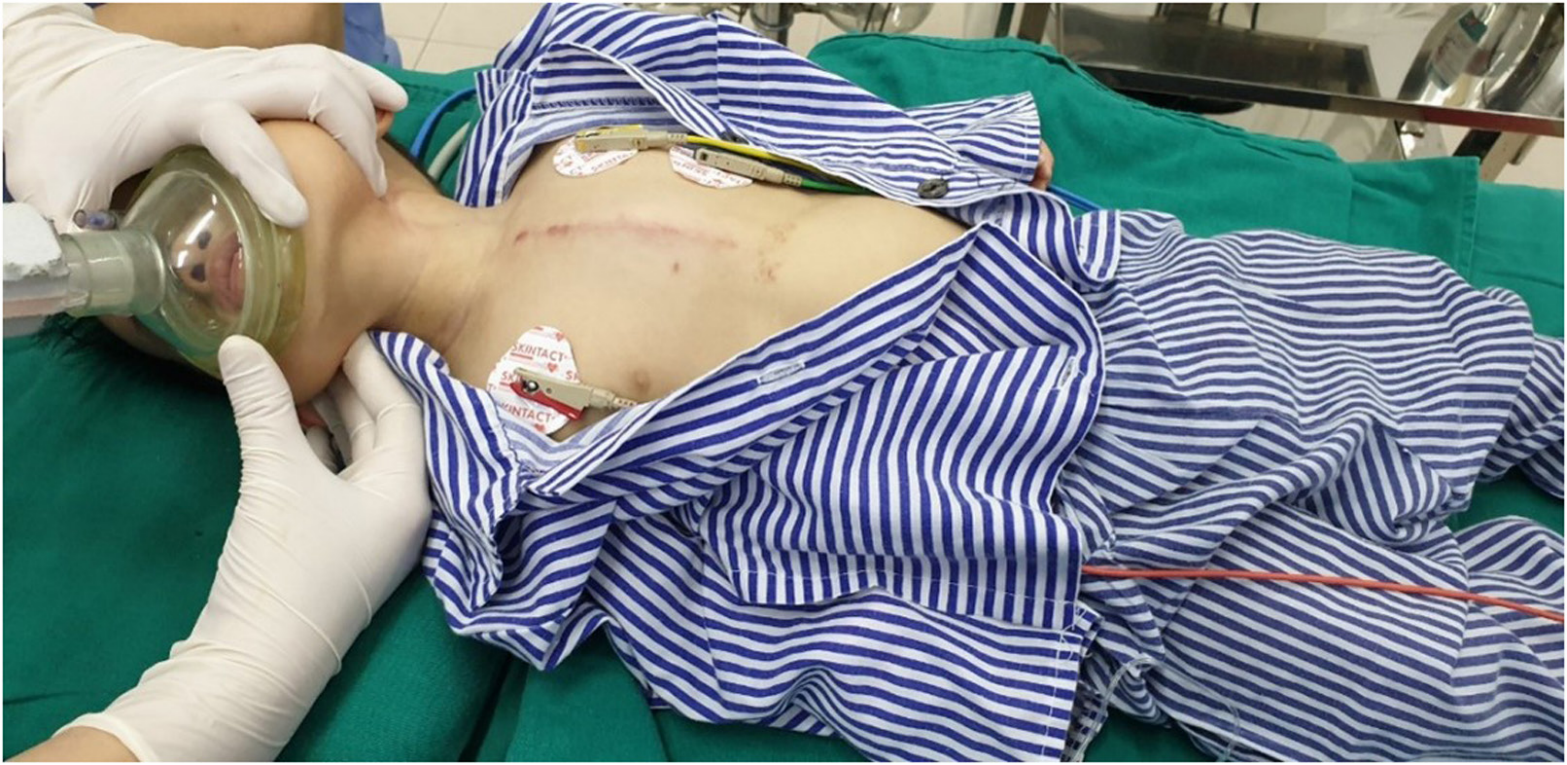
The patient before bronchoscopy intervention.

**FIGURE 3:**
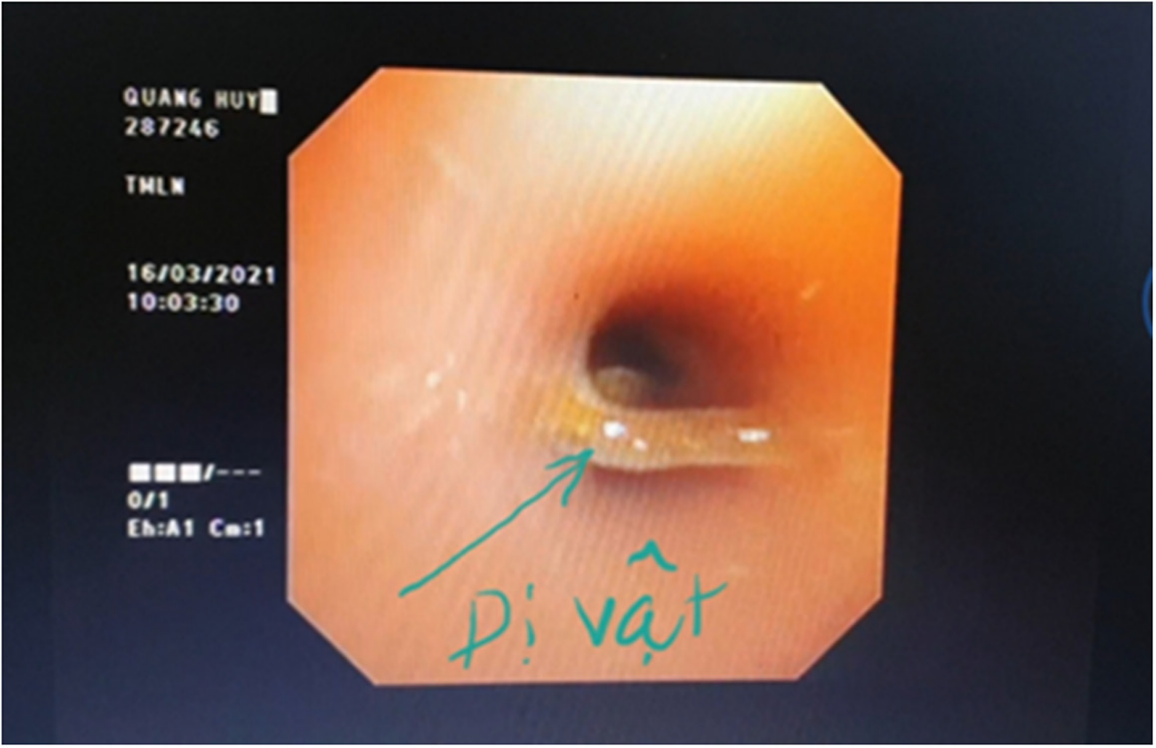
The foreign body in the right primary bronchus.

**FIGURE 4:**
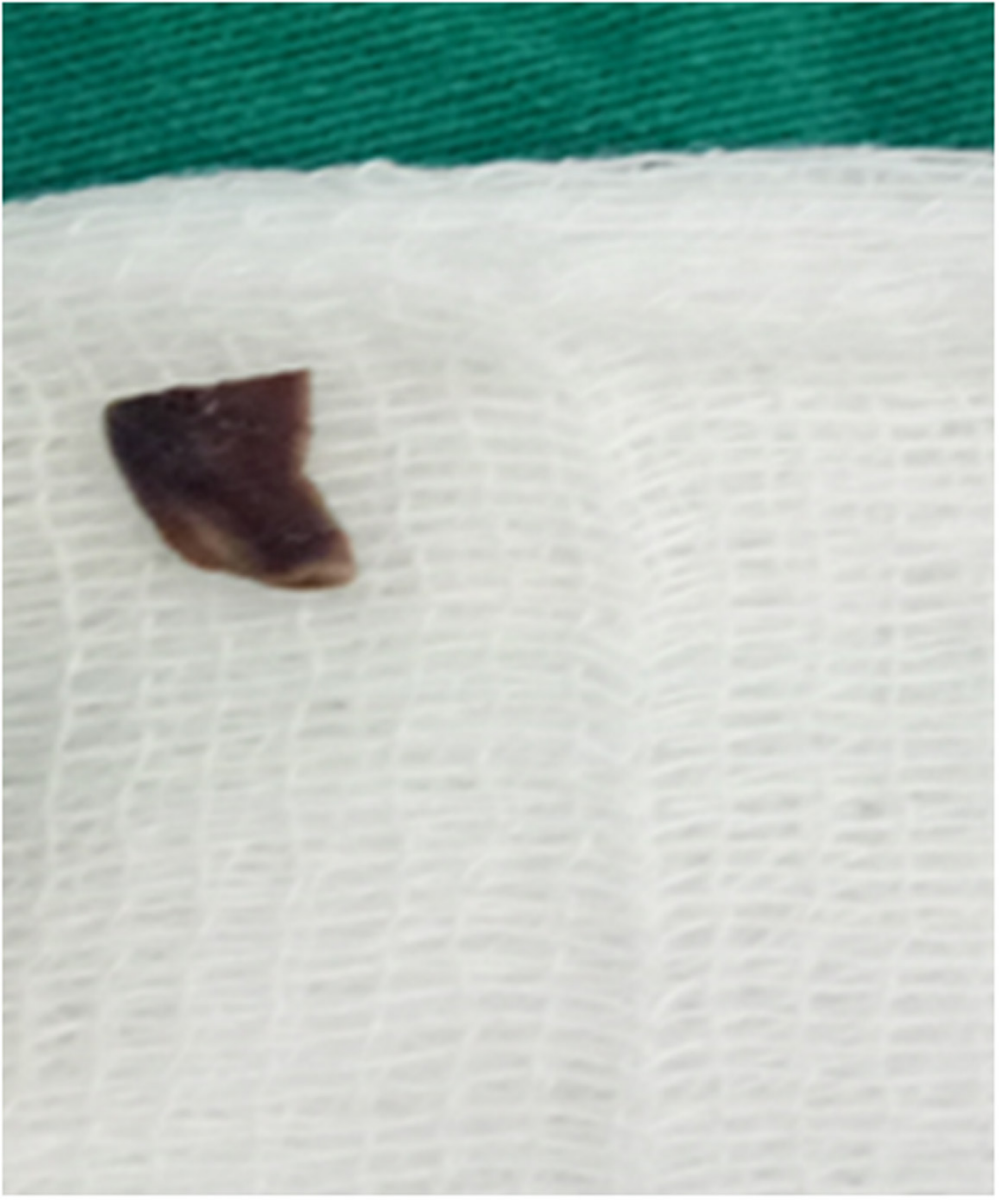
The sunflower seed removed from the bronchus.

We suspected the patient may have had a foreign body in the airway after 10 days of unsuccessful antibiotic therapy. We performed a flexible bronchoscopy for the patient, and during the procedure, we discovered a 0.5 × 1 cm sunflower seed lying across the right main bronchus (Figures 2, 3, 4).

**FIGURE 5:**
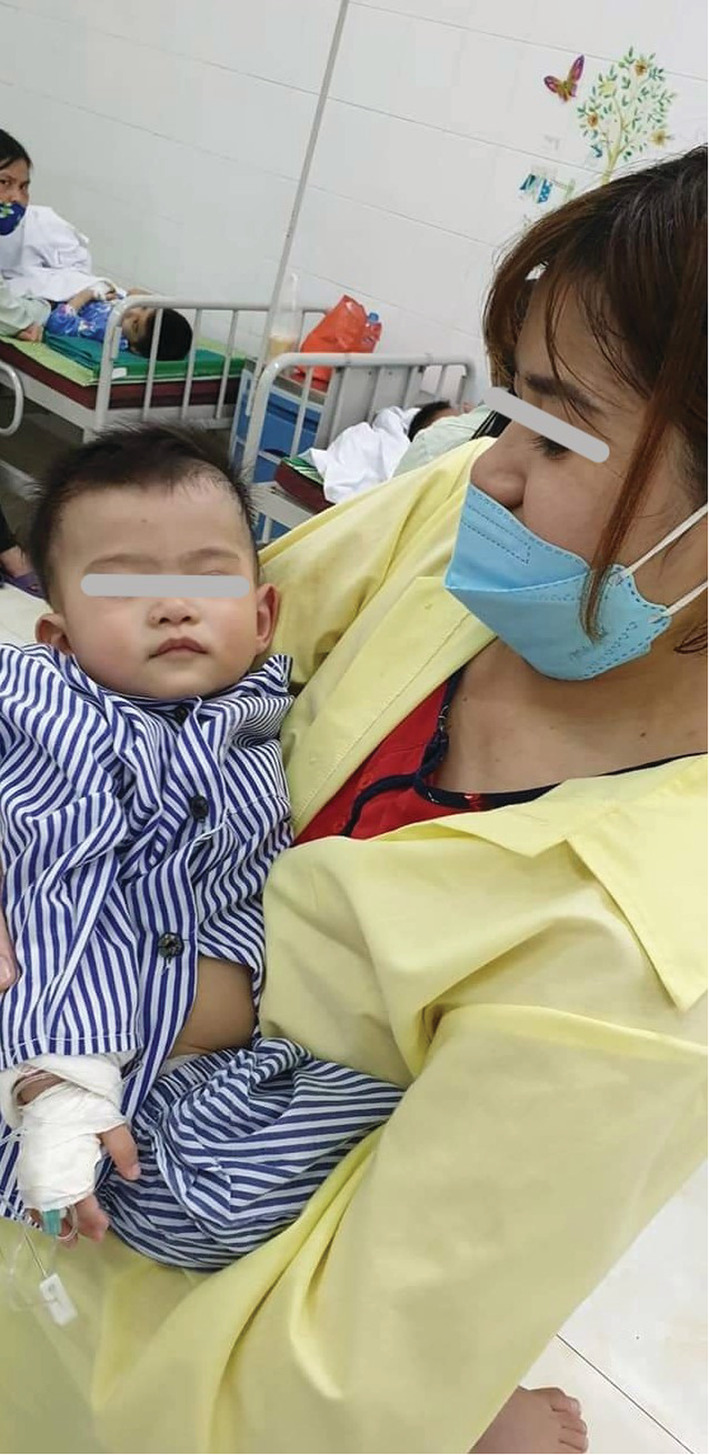
The patient recovered after the intervention.

After the intervention, his symptoms subsided, he ceased wheezing, coughing, and breathing problems, and his SpO_2_ level increased to 98%. He healed entirely and was released from the hospital after another 4 days of observation (Figure 5).

## 3 DISCUSSION

Foreign body aspiration is a typical emergency in children.^1^, ^2^, ^3^ Foreign bodies are usually of biological origin, typically nuts such as peanuts and sunflower seeds. However, the incidence of each foreign body depends on each region's geography and living conditions. A few cases of plastic and metal foreign bodies are reported, such as toys and pen tips.^1^

A child with a foreign body in the airway often experiences acute symptoms of penetration syndrome, including a violent coughing fit and sometimes sweating, which is the body's response to expel the foreign body. If the foreign body is still lodged in the respiratory tract, the child often has a prolonged productive cough, shortness of breath, and wheezing, depending on the object's size and the obstruction's location.^2^

Upon approaching a child with such symptoms, doctors elicit the child's eating history and other recent events to determine whether the child has inhaled a foreign body. However, taking this information in various cases is difficult, especially for infants, because they are unaware, and the family does not focus on or witness the event. Samarei's study in 2014 found that 13% of patients with foreign airway bodies did not remember events leading to inhaling the foreign body.^6^

Suppose doctors cannot find the critical history involving foreign body aspiration. In that case, it is prone to misdiagnose because the symptoms of a foreign body in the airway are similar to those of other respiratory tract diseases, including pneumonia, bronchiolitis, and other conditions. This is important in children with other risk factors such as asthma, recurrent pneumonia, a history of congenital heart disease, and cardiovascular surgery.^7^

Some studies have found that the rate of detecting airway foreign bodies on chest X‐rays is not high, about 32.2%–49%.^3^, ^8^ A CT scan can be more reliable and can be used to lower the rate of negative bronchoscopy.^9^, ^10^

Medical literature recorded many cases of foreign body aspiration. In a study in China, 35.4% of children with a foreign body in the airway were misdiagnosed, mainly mistaken for bronchiolitis. The primary cause is test errors, followed by failure or delay in eliciting critical history information.^11^

In this case, our patient suffered from coughing, wheezing, and shortness of breath. His chest X‐ray indicated no specific lesions. However, the patient showed quite similar symptoms to pneumonia, and he also had a history of surgery for tetralogy of Fallot, so it was very likely for doctors to misdiagnose.

Bronchoscopy is a standard method for diagnosing foreign bodies in the airway. Currently, bronchoscopy is the method of choice to remove a foreign body from the respiratory tract.^4^, ^5^

In this case, because the initial diagnosis was pneumonia, antibiotics treatment was indicated. However, we found that the symptoms did not regress. The patient still coughed a lot, had difficulty breathing, and wheezed, so we thought it might be a foreign body in the airway. Therefore, we decided to perform a flexible bronchoscopy and remove the foreign body.

The patient recovered perfectly and was discharged after 4 days of treatment in stable condition. However, the patient's family still cannot remember when he ate sunflower seeds.

## 4 CONCLUSION

Identifying foreign objects in children's airways could be challenging since patients frequently forget what their child has previously eaten. When approaching a child with wheezing, trouble breathing, or coughing, the clinicians should take a comprehensive medical history and consider the possibility of foreign body aspiration. For a precise diagnosis, a clinical examination and flexible bronchoscopy are recommended. When performing a bronchoscopy, it is essential to focus on foreign airway bodies, namely sunflower seeds, for timely removal.

## 5 RECOMMENDATION

When addressing a child with coughing and wheezing, it is essential to evaluate the possibility of a foreign body in the airway. Bronchoscopy is a reliable and effective method to diagnose and remove a foreign body timely. In addition, families should note what their children eat, and we should not let children eat sunflower seeds.

## AUTHOR CONTRIBUTIONS

SNN, TTV, BH, CVD, and HTN participated in the study design, protocol development and performance, data analysis, interpretation of data, writing of the manuscript, carrying out the clinical data collection and data analysis, and observing the patient during the treatment. TVT performed a bronchoscopy. All authors read and approved the final manuscript.

## CONFLICT OF INTEREST STATEMENT

The authors declare that they have no competing interests.

## ETHICAL APPROVAL

Approval for the study was obtained from the Medical Ethics Council of Haiphong University of Medicine and Pharmacy, and informed consent was obtained according to the Declaration of Helsinki.

## CONSENT

Written informed consent was obtained from the patient and his parents for the publication of these data and the accompanying images. A copy of the written consent is available for review by the Editor of this journal.

## Data Availability

The data that support the findings of this study are available from the corresponding author upon reasonable request.
